# Residual malaria transmission dynamics varies across The Gambia despite high coverage of control interventions

**DOI:** 10.1371/journal.pone.0187059

**Published:** 2017-11-02

**Authors:** Julia Mwesigwa, Jane Achan, Gian Luca Di Tanna, Muna Affara, Musa Jawara, Archibald Worwui, Majidah Hamid-Adiamoh, Fatoumatta Kanuteh, Sainey Ceesay, Teun Bousema, Chris Drakeley, Koen Peeters Grietens, Steve W. Lindsay, Jean-Pierre Van geertruyden, Umberto D’Alessandro

**Affiliations:** 1 Medical Research Council Unit The Gambia, Banjul, The Gambia; 2 Department of Global Health, Faculty of Medicine and Health Sciences, University of Antwerp, Antwerp, Belgium; 3 Centre for Primary Care and Public Health, Queen Mary University of London, London, United Kingdom; 4 West African Centre for Cell Biology of Infectious Pathogens, Department of Biochemistry, Cell & Molecular Biology University of Ghana, Accra, Ghana; 5 Department of Medical Microbiology, Radboud University Medical Center, Nijmegen, The Netherlands; 6 Faculty of Infectious and Tropical Diseases, London School of Hygiene and Tropical Medicine, London, United Kingdom; 7 Department of Public Health, Institute of Tropical Medicine, Antwerp, Belgium; 8 Amsterdam Institute for Social Science Research, University of Amsterdam, Amsterdam, The Netherlands; 9 School of Tropical Medicine and Global Health, Nagasaki University, Nagasaki, Japan; 10 School of Biological & Biomedical Sciences, Durham University, Durham, United Kingdom; Centro de Pesquisas Rene Rachou, BRAZIL

## Abstract

Over the last decades, malaria has declined substantially in The Gambia but its transmission has not been interrupted. In order to better target control interventions, it is essential to understand the dynamics of residual transmission. This prospective cohort study was conducted between June 2013 and April 2014 in six pairs of villages across The Gambia. Blood samples were collected monthly during the transmission season (June-December) from all residents aged ≥6 months (4,194 individuals) and then in April (dry season). Entomological data were collected monthly throughout the malaria transmission season. Ownership of Long-Lasting Insecticidal Nets was 71.5% (2766/3869). Incidence of malaria infection and clinical disease varied significantly across the country, with the highest values in eastern (1.7/PYAR) than in central (0.2 /PYAR) and western (0.1/PYAR) Gambia. Malaria infection at the beginning of the transmission season was significantly higher in individuals who slept outdoors (HR = 1.51, 95% CI: 1.02–2.23, p = 0.04) and in those who had travelled outside the village (HR = 2.47, 95% CI: 1.83–3.34, p <0.01). Sub-patent infections were more common in older children (HR = 1.35, 95% CI: 1.04–1.6, p <0.01) and adults (HR = 1.53, 95% CI: 1.23–1.89, p<0.01) than in younger children. The risk of clinical malaria was significantly higher in households with at least one infected individual at the beginning of the transmission season (HR = 1.76, p<0.01). Vector parity was significantly higher in the eastern part of the country, both in the south (90.7%, 117/129, p<0.01) and the north bank (81.1%, 227/280, p<0.01), than in the western region (41.2%, 341/826), indicating higher vector survival. There is still significant residual malaria transmission across The Gambia, particularly in the eastern region. Additional interventions able to target vectors escaping Long-Lasting Insecticidal Nets and indoor residual spraying are needed to achieve malaria elimination.

## Background

Over the last 15 years, the burden of malaria has decreased substantially worldwide, sub-Saharan Africa included, thanks to the scale up of standard control interventions such as prompt diagnosis and treatment, long-lasting insecticidal nets (LLINs), and indoor residual spraying (IRS) [1]. The Gambia is one of the first African countries in which such decline has been documented; between 2003 to 2007 the proportion of malaria positive slides decreased by 74%, a 4-fold decline, and malaria hospital admissions decreased by 81%, a 5-fold decline [2, 3]. These trends continued over the next few years, and between 2010 to 2015 malaria incidence and mortality declined by 60% [1]. Despite the observed improvement in malaria indicators and relatively high coverage of malaria control interventions (65% for LLINs [1]), malaria transmission, which is markedly seasonal (June-December), is still on-going and has become increasingly heterogeneous [4]. Such phenomenon has already been reported in other African countries [5, 6] at regional [7], village [8] and household level [9]. This observed heterogeneity may be due to diversity in transmission intensity, [9] and exposure [10, 11]. Variations in the ecology of the local vectors such as shift of biting time from late to early biting [12], increased outdoor biting behaviour [13] and changes in species composition [14, 15] have been shown to contribute to maintain transmission [16]. LLINs and IRS are effective against endophagic and endophilic vectors but may also select for exophagic, exophilic vectors [12]. As transmission further declines, pockets of transmission, possibly stable over time, will become increasingly important as countries target elimination [17].

In The Gambia, the observed malaria heterogeneity, i.e. increasing transmission from west to east, has been partly attributed to the variation in the distribution of the vector *An*.*(Anopheles) gambiae* complex *(sensu lato)*; *An*. *arabiensis* is found mainly in the eastern part of the country, *An*. *melas*, a brackish water species, in the central and western regions, and *An*. *gambiae* s.s. (*sensu stricto*) across the whole country [18]. *An*. *arabiensis* is predominantly outdoor biting and may contribute to maintaining outdoor transmission. Indeed, for individuals protected by LLINs, a small but important portion of vector feeding activity occurring at dawn and dusk accounts for approximately half of all transmission exposure to residual vector populations [19].

As transmission is markedly seasonal, it is assumed that malaria-infected but otherwise healthy individuals, i.e. without any clinical symptoms of malaria, have probably acquired their infections during the transmission season. In high transmission settings, microscopy can identify a substantial proportion of asymptomatic infections while in low transmission settings molecular methods are needed as most infection are sub-microscopic [20, 21]. Infected individuals can carry malaria infections for weeks or months, without any clinical manifestation [22], and restart the next malaria transmission season [23, 24].

National Malaria Control Programs (NMCP) in collaboration with academic institutions should estimate the magnitude of the problem of residual transmission, including information on human and vector behavior, and intervention effectiveness [25]. As the Gambia NMCP aims at achieving the pre-elimination status, i.e. slide positivity rate <5%, by 2020 [26], it is essential to understand the local residual transmission and the factors maintaining it, and to identify targets for new interventions.

## Methods

### Study sites

This cohort study was carried out in six sites across The Gambia, in the western, central and eastern part of the country, further subdivided into the north and south banks. This division corresponded roughly to five of the seven administrative regions, namely West Coast (WCR), North Bank (NBR), Lower River (LRR), Central River (CRR) and Upper River (URR) Regions, with the latter further divided in the north (URR-N) and south (URR-S) bank. In each of the study sites, we carried out a cross-sectional survey in November 2012, at the peak of the transmission season [4], in six villages located around the primary school with the highest prevalence of antimalarial antibodies as established by a nationwide school survey carried out in May 2012 [7]. In each site, the village with the lowest and the one with the highest malaria prevalence were selected with the aim of investigating local heterogeneity of transmission. This resulted in six village pairs; each pair of villages were approximately 1–3 km apart, with populations ranging between 100 and 700 individuals.

### Recruitment and follow-up

In April 2013, following village sensitization meetings, all residents (as confirmed by household heads), aged ≥6 months were recruited after provision of individual informed consent (parents for children) and assent for children ≥12 years old. Individuals with chronic medical conditions were also included. Recruitment lasted three months (May-July 2013) to maximize coverage. Case record forms (CRFs) were pre-tested in March 2013, prior to starting the study, to evaluate the logical flow of the questions and correct interpretation of each question into the local language by the field coordinator and the understanding of the questions by the interviewees S1 File Case record form. The pre-testing was conducted in three rounds in a village near the study site in the western region. During each round, the senior study field coordinators and a nurse administered the questionnaire to ten residents. The CRFs were also reviewed by the data manager. The lessons learnt from the pre-test were used to change the CRFs prior the finalization of the data base and field work implementation. Demographic data and LLIN household coverage were collected at baseline.

Monthly surveys, in which all residents present in the villages were included, were carried out during the last 2 weeks of each month, between June and December 2013; an additional survey was done in April 2014, during the dry season. At each survey, information on the use of LLIN, sleeping habits (outdoors), malaria symptoms, antimalarial treatment, and travel history was collected. Village health workers, one per village, helped the study nurses by providing information on participants who travelled, or migrated out of the village or had died. Axillary temperature was measured using a digital thermometer. A blood sample was collected by finger prick for haemoglobin measurement, malaria diagnosis by microscopy and molecular analysis on dry blood spots filter paper, (Whatman 3 Corporation, Florham Park, NJ, USA). Clinical malaria patients were identified by passive case detection at local health facilities or in villages by study nurses, and were treated with artemether-lumefantrine. Clinical malaria was defined as a history of fever in the previous 24 hours and / or axillary temperature ≥ 37.5°C with a positive Rapid Diagnostic Test (RDT) (Paracheck Pf, Orchid Biomedical System, India). Human behaviour was assessed by asking about travel history outside the village and sleeping outdoors at night in the previous 30 days. A household was defined as a group of individuals living in the same building and eating from the same pot. The structure of each house was defined as traditional or modern based on the following characteristics; type of roof (metal/cement or grass thatched), type of wall (mud or cement), open or closed eaves, presence of windows and presence or absence of screens on the windows. The proportion of houses with each of these variables was defined as the number of houses with that specific variable divided by the total number of houses in each village. LLIN use, sleeping outdoors at night, travel outside the village were defined as the number of individuals that reported LLIN use the previous night, sleeping outdoors and travelling any time during the month by the total number of individuals surveyed, respectively. *P*. *falciparum* infections were detected by diagnostic nested PCR from the collected dry blood spots. Asymptomatic malaria infections were defined as positive nested PCR in individuals without fever (or other acute symptoms), and no history of recent antimalarial treatment [27]. Sub-patent infections were infections detected by nested PCR but negative by microscopy in asymptomatic individuals.

### Molecular diagnostics and parasitology

For diagnostic nested PCR, three 6mm dried filter paper blood spots (DBS) were punched into a 96-well plate. DNA was extracted from the dry blood spots using the automated QIAxtractor robot (Qiagen). Negative and positive (3D7) controls were included to control for cross-contamination and DNA extraction efficiency, respectively. The blood spots were lysed by incubating them in tissue digest buffer at 60°C for 1 hour and digested eluates were applied onto capture plates, washed, and the DNA eluted into 80μl.The extracted DNA (4μl) was used in a nested PCR amplifying the multi-copy Plasmodium ribosomal RNA gene sequences using genus and species specific primers [28, 29]. All PCR products were run using the QIAxcel capillary electrophoresis system (Qiagen), using the screening cartridge and 15–1000 bp-alignment markers. Results were exported and double scored using both the QIAxcel binary scoring function and manually by visualization of the gel images; discrepancies were scored by a third independent reader [30]. Positive nested PCR samples (1,864) were selected for microscopy and a random sample of them (1,442) for gametocyte detection by mRNA Quantitative Nucleic Acid Sequence based Amplification (QT-NASBA) [31].

Thick blood smears were stained with 2% Giemsa for 30 minutes and examined by two independent microscopists. Discrepancies were settled by a third reader. Parasite density was estimated by counting the number of parasites per 200 WBC, and assuming 8,000 WBC/μL of blood. Blood smears were considered negative if no parasites were found after reading 100 high power fields.

### Entomological surveys

Monthly entomological sampling was done from 7pm to 7am by human landing catches (HLCs) and CDC light trap catches (LTC). Indoor and outdoor HLCs were conducted in two houses per village for three consecutive nights (n = 6). Upon completion of the HLCs, LTCs were set up in six houses per village on alternative nights for two nights (n = 12). Each morning, mosquitoes were counted and species identified morphologically. The *An*. *gambiae sensu lato* (*s*.*l*) mosquitoes were separated from *An*. *funestus* and stored in separate tubes with silica gel. Other anophelines and culicine mosquitoes were counted and discarded. *An*. *gambiae s*.*l*. females captured by HLCs were dissected to extract the ovaries and determine parity [32, 33]. *An*. *gambiae s*.*l*. head and thorax samples were used for the detection of *P*. *falciparum* circumsporozoite protein (CSP) by ELISA [34].

### Study outcomes

The primary outcome was incidence of *P*. *falciparum* infection (malaria infection) as determined by nested PCR.

Secondary outcomes included; incidence and prevalence of clinical malaria, prevalence of asymptomatic malaria infections, gametocytes, and sub-patent infections.

Entomological outcomes included; entomological inoculation rate (EIR) and vector parity.

### Statistical analysis

Data were double entered into a Microsoft Access^®^ database and analysed using Stata 14 (College Station, Texas, USA). Recurrent episodes of *P*. *falciparum* infection were categorized as 1, 2, and ≥ 3 episodes per individual. Anaemia was categorized as mild, moderate or severe according the WHO age-specific criteria [35]. Binary variables were summarized into proportions with the 95% confidence intervals. The two sample test of proportions was used to compare proportions of secondary outcomes between the WCR and each of the other regions (NBR, LRR, CRR, URR-S, and URR-N) and between July and each of the other months (August, September, October, November and December). The Benjamin-Hochberg procedure was used to correct for multiple testing because several tests for proportions were carried out. Pearson’s chi-squared test was used to determine outcomes’ trends during the transmission season.

Monthly incidence rates of *P*. *falciparum* infection were calculated by dividing the number of new infections (positive individuals who were nested PCR negative the previous monthly survey) by person-years. Incidence rate ratios (IRRs) compared the incidence of *P*. *falciparum* infection and clinical malaria between WCR and the other regions (NBR, LRR, CRR, URR-S, and URR-N). A Cox proportional hazards model and Kaplan-Meier survival curves were used to estimate the risk of clinical malaria among members within the same household with or without at least a malaria-infected individual at the start of the transmission season (June-July). Cox proportional hazards models with a shared frailty at region level and controlled for changes in variables (e.g. LLIN use) over time were used to predict the factors associated with *P*. *falciparum* infection at the start of the transmission season (June-July) and factors associated with sub-patent infection. A Poisson model was used to predict individual’s and household’s risk factors of recurrent malaria infections. Confidence intervals for gametocyte prevalence were calculated by the Wilson Score method with continuity correction. The sporozoite rate was the proportion of *P*. *falciparum* CSP positive mosquitoes divided by the total number of mosquitoes caught. The human-biting rate (HBR) was expressed as the number of bites per person per night (b/p/n) determined by dividing the number of mosquitoes collected by the number of volunteers per night. The entomological inoculation rate (EIR) was estimated by multiplying the sporozoite rate by the human-biting rate [33]. Vector densities by HLC were weighed according to the proportion of individuals sleeping both indoors and outdoors and estimated by month and region. The weighted proportions were then compared between WCR and the other regions, and across the transmission season.

Parity was defined as the proportion (p) of parous vectors divided by the total collected by HLC and LTC (p/np+p), by region. Pairwise Binary data between vector parity and incidence of malaria infection were compared using a Pearson chi-squared test and the correlations were based on the Spearman rank based approach.

### Ethical approval

Verbal consent from the study communities was obtained at village meetings prior to field activities. Written informed consent was obtained from all participants ≥ 18 years old and assent from children aged 11–17 years. Parents/guardians provided written consent for children <18 years. All households selected for LTCs and volunteers for HLCs provided additional written informed consent. The study was approved by the Scientific Coordinating Committee of the Medical Research Council Unit The Gambia (SCC 1318), and the Gambia Government/MRC Joint Ethics Committee.

## Results

**Fig 1:**
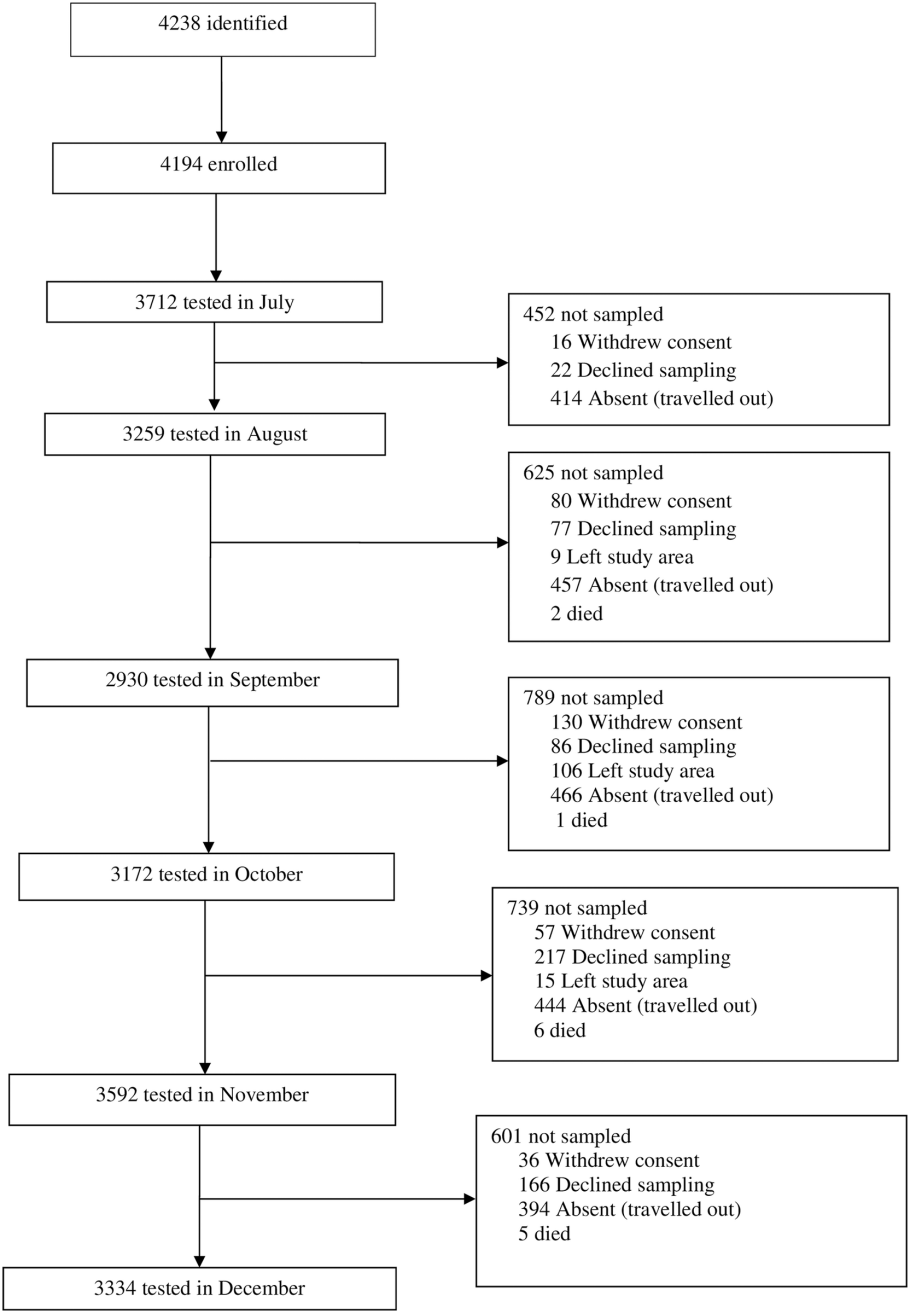
Study flow chart. The total follow-up time was 1,592.5 person years at risk (PYAR). The median age was 13 years (Inter-quartile range (IQR) 5, 28; range 6 months-90 years). LLINs ownership was high (71.49%, 2766/3869) and their use varied significantly by month, with July, at the start of the transmission season, having the lowest proportion (52.22%, 1916/3669), and increasing significantly from August (87.05%, 2811/3229) to October (94.04%, 2809/2987), p<0.01 (Fig 2).

In June 2013, a total of 4,194 residents were enrolled from 426 households across the twelve study villages (Fig 1)

Sleeping outdoors was high in July (42.11%, 1540/3657) but declined significantly in September (3.15%, 89/2826, p<0.01) and November (1.55%, 52/3355, p<0.01) (Table 1).

**Table 1:** Study participant and house structural characteristics in the study sites.

Variable	Frequency (%)
**Gender (N = 4,193)**	
Male	1,932 (46.08)
Female	2,261 (53.92)
**Age categories (N = 4,171)**	
6 months to 5 years	834 (19.99)
> 5 to 15 years	1388 (33.27)
>15 to 30 years	928 (22.24)
> 30 years	1021 (24.47)
**Anaemia (Hb<10 g/dl) July 2013**	
Children ≤ 5 years (N = 931)	218 (23.41)
Children 5 to <15 years (N = 1048)	150 (14.31)
Women 15 to 45 years (N = 759)	231 (30.43)
**LLIN ownership (N = 3,869)**	2,766 (71.49)
**Slept under LLIN the previous night**	
June (N = 1,514)*	781 (51.58)
July (N = 3,669)	1,916 (52.22)
August (N = 3,229)	2,811 (87.05)
September (N = 2,860)	2,457 (85.91)
October (N = 2,987)	2,809 (94.04)
November (N = 3,374)	2,625 (77.80)
December (N = 3,290)	1,988 (60.43)
**Sleep outdoors at night**	
June (N = 1,486)*	211 (14.19)
July (N = 3,658)	1,540 (42.09)
August (N = 3,288)	132 (4.0)
September (N = 2,826)	89 (3.15)
October (N = 3,211)	223 (6.94)
November (N = 3,355)	52 (1.55)
December (N = 3,286)	728 (22.15)
**Travel outside the village (N = 20,768)**	2,569 (12.36)
**Roof structure (N = 426)**	
Cement/concrete	5 (1.17)
Metallic/corrugates iron sheets	315 (73.94)
Grass thatched	106 (24.88)
**Wall structure (N = 423)**	
Cement plastered	79 (18.67)
Mud bricks	344 (81.32)
**Eaves present (N = 423)**	349 (82.51)
**Window screens (N = 347)**	16 (4.61)

*URR-S and URR-N not sampled in June

At the start of the transmission season, in June and July, the overall malaria prevalence was 4.94% (76/1537, 95% CI: 4.26–6.62) and 4.86% (181/3723, 95% CI: 4.57–6.07), respectively. At this time, the risk of malaria infections was significantly lower in females (Hazard Ratio (HR) = 0.72, 95% CI: 0.54–0.97, p = 0.03) than males, among LLIN owners (HR = 0.49, 95% CI: 0.34–0.69, p <0.01), than in individuals without LLINs and lower in individuals sleeping under an LLIN (HR = 0.32, 95% CI: 0.19–0.54, p<0.01); it was significantly higher among those who had travelled outside the village (HR = 2.48, 95% CI: 1.83–3.34, p <0.01) and in those who slept outdoors (HR = 1.51, 95% CI: 1.02–2.23, p = 0.04). (Table 2)

**Table 2:** Risk factors of *P*. *falciparum* infection at the start of the transmission season.

	Univariate analysis		Multivariate analysis	
	Hazard ratio (95% CI)	P value	Hazard ratio (95% CI)	P value
**Age groups**				
< 4.9 years	1		1	
5–14.9 years	1.32 (0.81–2.15)	0.26	1.25 (0.77–1.99)	0.36
15–29.9 years	0.91 (0.75–1.10)	0.32	0.82 (0.60–1.12)	0.21
≥ 30 years	0.83 (0.57–1.11)	0.21	0.73 (0.49–1.07)	0.11
**Gender**				
Males	1		1	
Females	0.69 (0.57–0.84)	<0.01	0.72 (0.54–0.97)	0.03
**LLIN ownership at the start of season**				
No	1			
Yes	0.43 (0.30–0.61)	<0.01	0.49 (0.34–0.69)	< 0.01
**Slept under LLIN during the transmission season**				
No	1			
Yes	0.22 (0.1–0.5)	<0.01	0.32 (0.19–0.54)	<0.01
**Travelled outside the village**				
No	1			
Yes	5.59 (2.4–12.9)	<0.01	2.48 (1.83–3.34)	<0.01
**Sleep outdoors**				
No	1			
Yes	4.42 (1.7–11.3)	0.002	1.51 (1.02–2.23)	0.04

**Fig 2:**
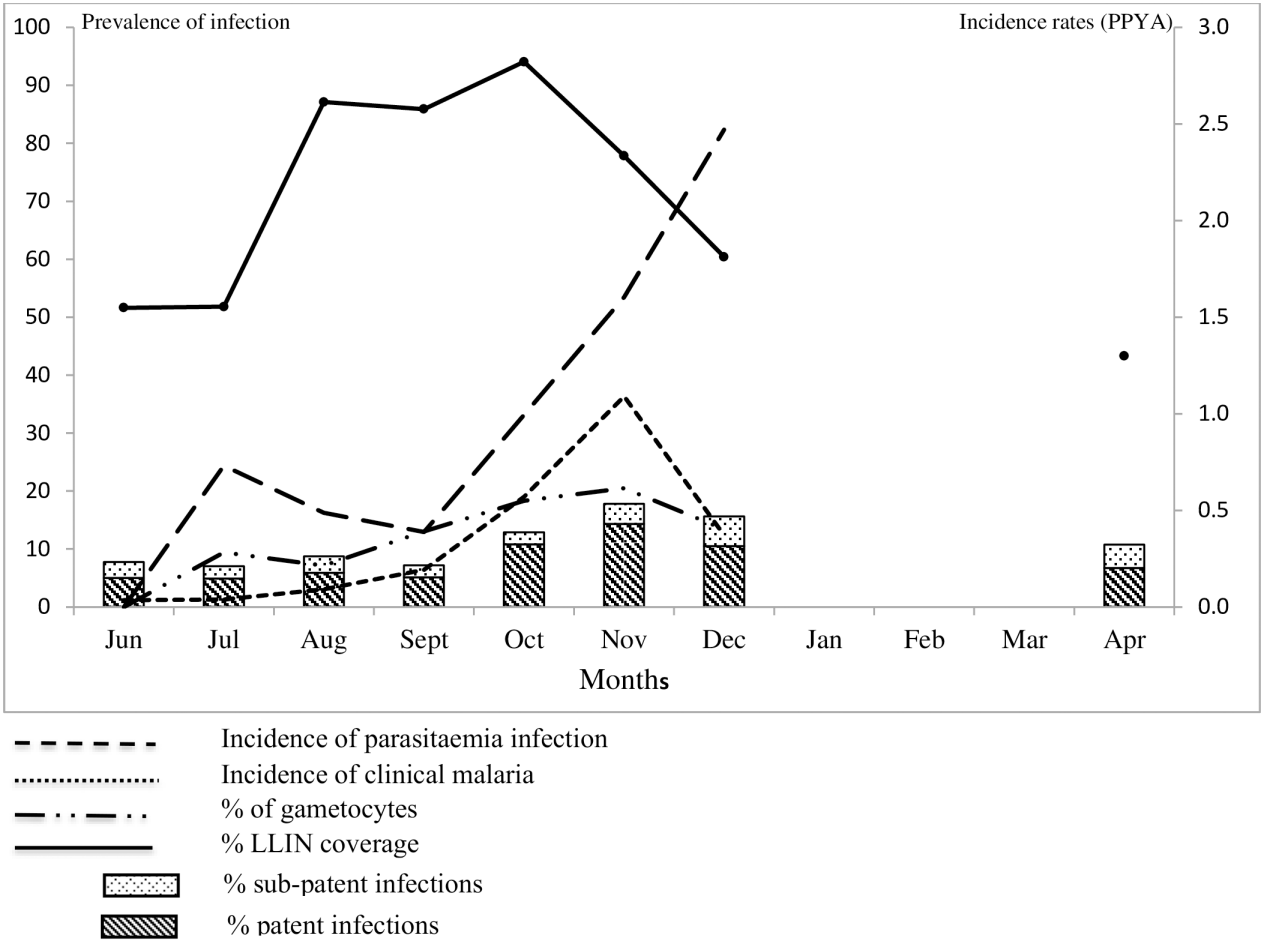
Overall prevalence and incidence of malaria infection, clinical malaria, gametocytaemia and proportion of LLIN use by month.

During the entire transmission season, about a third (34.98%, 644/1841) of infections were sub-patent, the highest proportion found in June (55.26%, 42/76) and July (43.09%, 78/181), at the beginning of the transmission season, while the lowest was in October (19.15%, 68/355) (Fig 2). Sub-patent infections were significantly more frequent in June than September (40.26%, 62/154 p = 0.03), October (19.15%, p<0.01) and November (24.38%, 127/521 p<0.01). Older children (HR = 1.35, 95% CI: 1.04–1.6, p = 0.01) and adults (HR = 1.53, 95% CI: 1.23–1.89, p<0.01) had a higher risk of sub-patent infections compared to younger children. Similarly, individuals with mild anaemia (HR = 1.4, 95% CI: 1.23–1.6, p = 0.01) had a higher risk of having sub-patent infections compared to those with normal haemoglobin levels. (Table 3)

**Table 3:** Risk factors of sub-patent *P*. *falciparum* infections*.

	Univariate analysis		Multivariate analysis	
Variable	HR (95% CI)	p value	HR (95% CI)	p value
**Age category**				
< 4.9 years	1		1	
5–15 years	1.39 (1.07–1.81)	0.01	1.35 (1.04–1.6)	0.01
16–30 years	1.49 (1.25–1.78)	< 0.01	1.53 (1.23–1.89)	< 0.01
≥30 years	1.3 (0.9–1.89)	0.07	1.47 (0.9–1.91)	0.1
**Gender**				
Males	1		1	
Female	0.78 (0.7–1.15)	0.31	0.79 (0.56–1.13)	0.1
**Anaemia**				
Normal	1		1	
Mild	1.4 (1.2–1.6)	< 0.01	1.4 (1.23–1.6)	< 0.01
Moderate	1.3 (0.9–1.7)	0.1	1.3 (0.9–1.7)	0.1
Severe	1.2 (0.8–1.9)	0.3	1.3 (0.8–2.0)	0.2
**LLIN use at night**				
No	1			
Yes	0.9 (0.6–1.6)	0.21	-	-
**Sleep outdoors**				
No	1			
Yes	0.9 (0.5–0.80)	0.8	-	-
**Travelled outside the village**				
No	1			
Yes	1.4 (0.7–2.9)	0.38	-	-

* Sub-patent infections: positive by nested PCR and negative by microscopy in asymptomatic individuals

Malaria prevalence during the dry season (April 2014) was 6.68% (212/3173), and 60.37% (128/212) of these infections were sub-patent. Most infected individuals in April (74.53%, 158/212) were negative in December 2013.

The overall incidence rate of malaria infections was 1.01 infections/PYAR (95% CI: 0.94–1.17) and that of clinical malaria was 0.38 infections/PYAR (95% CI: 0.36–0.43). Incidence of malaria infections was low in July (0.73/PYAR; 95% CI: 0.62–0.86), decreased significantly in August (0.49/PYAR; 95% CI: 0.24–0.57) and September (0.39/PYAR; 95% CI: 0.32–0.46), p<0.01, and increased steadily in October (0.99/PYAR; 95%CI: 0.89–1.11), November (1.60/PYAR; 95%CI: 1.46–1.76) and December (2.47/PYAR; 95% CI: 2.21–2.77) (p<0.01). (Table 4)

**Table 4:** Incidence rates of *P*. *falciparum* infections by month (95%CI).

Month	*P*. *falciparum* episode*s*	Person-years (PYAR)	Incidence rates	Incidence rate ratio	p value
July	151	206.76	0.73 (0.62–0.86)	1	
August	156	320.71	0.49 (0.42–0.57)	0.67 (0.53–0.84)	<0.01
September	121	312.19	0.39 (0.32–0.46)	0.53 (0.42–0.68)	<0.01
October	314	316.11	0.99 (0.89–1.11)	1.36 (1.12–1.67)	0.01
November	453	282.48	1.60 (1.46–1.76)	2.20 (1.83–2.67)	<0.01
December	300	121.41	2.47 (2.21–2.77)	3.39 (2.79–4.16)	<0.01

* Incidence rate ratios comparing incidence in July with the other months during the transmission season

Similarly, the incidence of clinical malaria increased significantly from August (0.09/PYAR, 95% CI: 0.06–0.13) to December (0.37/PYAR, 95% CI: 0.28–0.49) with the peak in November (1.09/PYAR; 95% CI: 0.98–1.20) (p<0.01), when compared to July. (Table 5 and Fig 2).

**Table 5:** Incidence rate of clinical malaria by month (95%CI)*.

Month	Clinical malaria episodes	Person-years (PYAR)	Incidence rates	Incidence rate ratio	p value
July	8	206.76	0.04 (0.02–0.08)	1	
August	29	320.71	0.09 (0.06–0.13)	2.34 (1.05–5.94)	0.003
September	60	312.19	0.19 (0.15–0.25)	4.98 (0.37–12.05)	<0.01
October	180	316.11	0.57 (0.49–0.66)	14.74 (7.32–34.66)	<0.01
November	308	282.48	1.09 (0.98–1.20)	28.26 (14.16–66.05)	<0.01
December	45	121.41	0.37 (0.28–0.49)	9.62 (4.49–23.64)	<0.01

* Incidence rate ratios comparing incidence rates in July to other months during the transmission season

Malaria prevalence in July was similar and not statistically different across the regions (2.84% in WCR, 3.41% in NBR, 4.70% in LRR, 3.53% in CRR), with the exception of the URR-S (5.83%, 95% CI: 4.45–7.20, p = 0.05) and URR-N (8.85%, 37/418, 95%CI: 6.31–11.39, p<0.01) where it was significantly higher than in the WCR.

**Fig 3:**
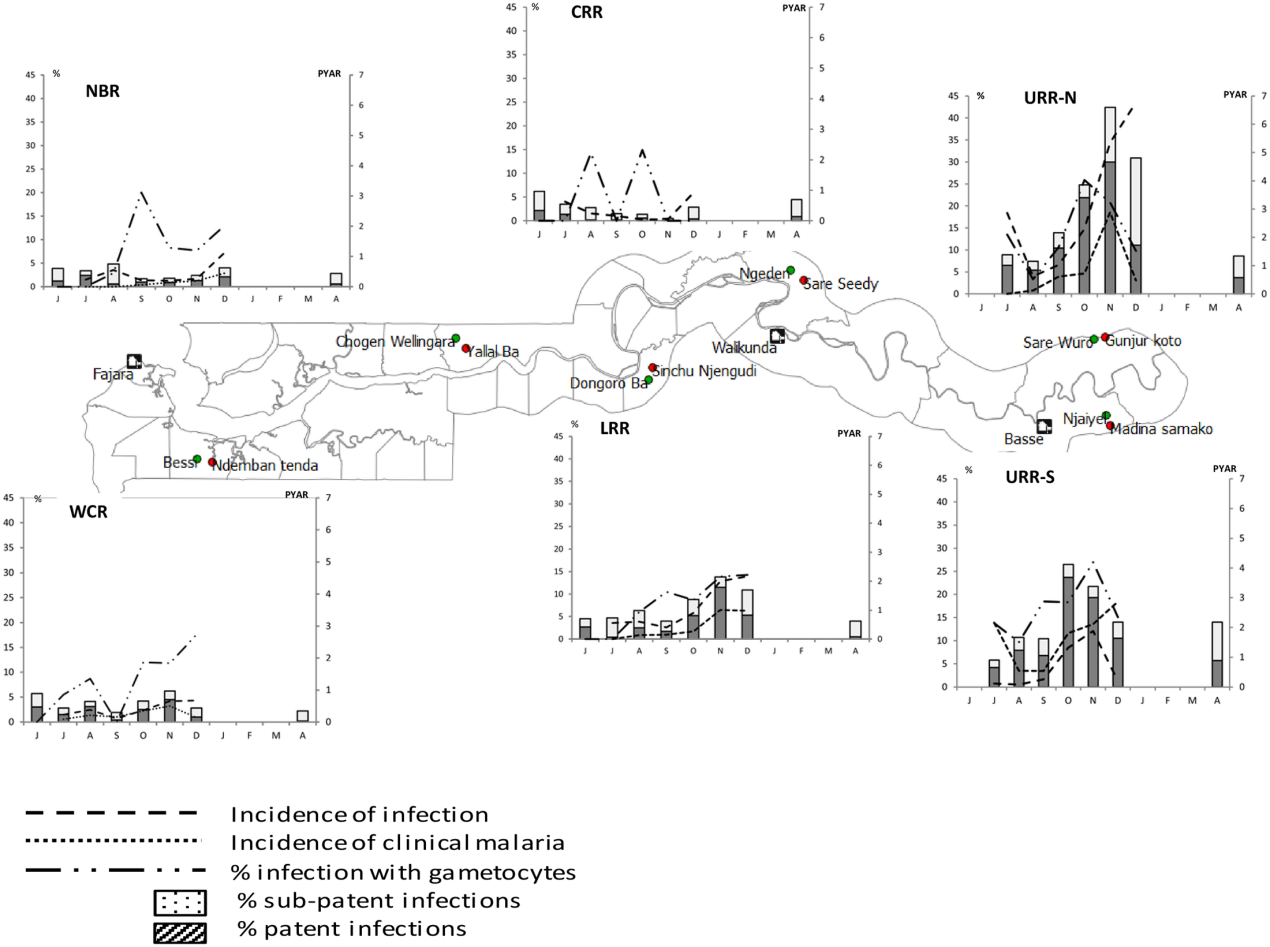
Prevalence and incidence of malaria infection and clinical malaria by month and region. The incidence rates were significantly higher in three regions compared to the WCR; URR-N (*P*. *falciparum* infection = 2.82/PYAR, 95% CI: 2.57–3.10; and clinical malaria = 1.0/PYAR, 95%CI: 0.82–1.11, p<0.01) and URR-S (*P*. *falciparum* infection = 1.42/PYAR, 95% CI: 1.31–1.54; and clinical malaria = 0.8/PYAR, 95%CI: 0.68–0.85; p<0.01) had the highest incidence, followed by LRR (*P*. *falciparum* infection = 0.98/PYAR, 95% CI:0.86–1.12; clinical malaria = 0.36/PYAR, 95%CI: 0.29–0.455; p<0.01). The incidence of *P*. *falciparum* infections in the WCR (0.47/PYAR, 95% CI: 0.40–0.56) was 2 times higher than in the CRR (0.29/PYAR, 95%CI: 0.22–0.37) (p<0.01) where not a single clinical malaria case was detected (Table 6 and Fig 3).

The pattern of both *P*. *falciparum* infection and clinical malaria incidence during the transmission season differed substantially between regions (Fig 3)

**Table 6:** Incidence of infection and clinical malaria per person-years, by month and region (95%CI).

*P*. *falciparum* infection						
Regions	WCR	NBR	LRR	CRR	URR-S	URR-N
July	0.23	0.24	0.56	0.62	-	-
	(0.13–0.39)	(0.13–0.42)	(0.37–0.85)	(0.39–1.01)		
August	0.38	0.55	0.60	0.24	0.56	0.62
	(0.25–0.57)	(0.38–0.80)	(0.41–0.89)	(0.13–0.43)	(0.42–0.74)	(0.44–0.96)
September	0.11	0.21	0.41	0.16	0.54	1.03
	(0.05–0.24)	(0.11–0.38)	(0.26–0.67)	(0.13–0.43)	(0.40–0.72)	(0.74–1.44)
October	0.37	0.20	0.91	0.04	1.81	2.30
	(0.24–0.57)	(0.11–0.38)	(0.66–1.26)	(0.01–0.18)	(1.55–2.12)	(1.85–2.87)
November	0.65	0.26	2.0	0.05	2.11	5.35
	(0.47–0.92)	(0.15–0.46)	(1.58–2.53)	(0.01–0.21)	(1.81–2.47)	(4.61–6.22)
December	0.67	1.13	2.17	0.93	2.87	6.77
	(0.40–1.13)	(0.73–1.76)	(1.59–2.96)	(0.51–1.67)	(2.36–3.50)	(5.63–8.14)
IRR compared to WCR		0.4	0.6	2.1	0.4	0.2
		(0.3–0.5)	(0.5–0.7)	(1.7–2.7)	(0.3–0.5)	(0.15–0.24)
		p<0.01	p<0.01	p<0.01	p<0.01	p<0.01
**Clinical malaria**						
July	0.08	0	0	0	0.13	-
	(0.04–0.21)				(0.04–0.39)	
August	0.21	0	0.14	0	0.08	0.12
	(0.11–0.36)		(0.02–0.22)		(0.04–1.65)	(0.05–0.33)
September	0.16	0.06	0.15	0	0.26	0.61
	(0.08–0.31)	(0.02–0.18)	(0.07–0.33)		(0.16–0.38)	(0.37–0.92)
October	0.35	0.14	0.27	0	1.32	0.72
	(0.23–0.55)	(0.07–0.29)	(0.13–0.47)		(1.11–1.62)	(0.42–0.98)
November	0.50	0.19	1.01	0	1.89	2.88
	(0.34–0.73)	(0.09–0.35)	(0.67–1.36)		(1.42–2.07)	(2.36–3.68)
December	0.14	0.45	0.98	0	0.21	0.47
	(0.04–0.44)	(0.27–0.99)	(0.61–1.64)		(0.10–0.51)	(0.23–1.14)
IRR compared to WCR		2.3	0.7	0	0.4	0.25
		(1.8–3.0)	(0.5–0.7)		(0.3–0.5)	(0.2–0.3)
		p<0.01	p = 0.002		p<0.01	p<0.01

The proportion of sub-patent infections varied substantially across regions, and with the highest values in CRR (74.23%, 72/97) and NBR (62.86%, 88/140) and the lowest in URR-N (35.39%, 200/565) and URR-S (24.47%, 195/797) (Fig 3). Sub-patent infections occurred significantly less in the WCR (22.28%, 85/184) (p<0.01) than in the other regions, with the exception of URR-S (p = 0.53).

None of the infections identified in June carried gametocytes; however, the proportion of infections with gametocytes increased significantly, from 9.39% (17/181) in July, to 21.69% (113/521 p<0.01) in November (Fig 2) (S1 Table). During the dry season, the overall proportion of infections with gametocytes was 14.1% (31/220).

Most individuals (17.99%, 752/4179) had a single episode of *P*. *falciparum* infection; 5.36% (224/4179) had two and 2.46% (103/4179) had three or more episodes. Multiple episodes of malaria infections were significantly more frequent in the URR-N (25.54%, 118/462) and URR-S (10.65%, 123/1154) than in WCR (2.01%, 15/747) (p<0.01). Older children (5 to 14.9 years) (IRR = 1.42, 95%CI: 1.21–1.59, p<0.01) had a higher risk of multiple episodes and adults ≥30years a lower risk (IRR = 0.75, 95% CI: 0.63–0.89, p = 0.002) than younger children. Similarly, females (IRR 0.87, 95% CI: 0.78–0.97, p = 0.02) and LLIN owners (IRR = 0.84, 95% CI: 0.74–0.94, p = 0.003) had a lower risk of multiple episodes of infection (Table 7).

**Table 7:** Risk factors for ≥ 2 episodes of malaria infection.

	Poisson model		Multilevel Poisson model (random intercept for regions)	
	IRR*	p value	IRR*	p value
**Age groups**				
< 4.9years	1		1	
5–14.9years	1.42 (1.31–1.53)	<0.01	1.39 (1.21–1.59)	< 0.01
15–29.9years	1.01 (0.85–1.21)	0.9	0.89 (0.76–1.06)	0.21
≥30years	0.73 (0.58–0.91)	0.01	0.75 (0.63–0.89)	0.002
**Gender**				
Males	1		1	
Females	0.81 (0.75–0.88)	< 0.01	0.87 (0.78–0.97)	0.02
**Hb**				
Normal	1		1	
Mild anaemia	1.16 (1.05–1.28)	0.004	1.12 (0.98–1.29)	0.09
Moderate anaemia	1.01 (0.90–1.13)	0.84	1.02 (0.87–1.19)	0.85
Severe anaemia	1.12 (0.76–1.64)	0.58	0.90 (0.64–1.28)	0.57
**LLIN ownership**				
No	1		1	
Yes	0.62 (0.42–0.91)	0.01	0.84 (0.74–0.94)	0.003
**Sleep outdoors**				
No	1		1	
Yes	1.28 (0.94–1.74)	0.12	1.02 (0.83–1.24)	0.88
**Travelled outside the village**				
No	1		1	
Yes	1.33 (0.9–1.89)	0.10	0.92 (0.75–1.14)	0.46

*IRR: Incidence Rate Ratio

**Fig 4:**
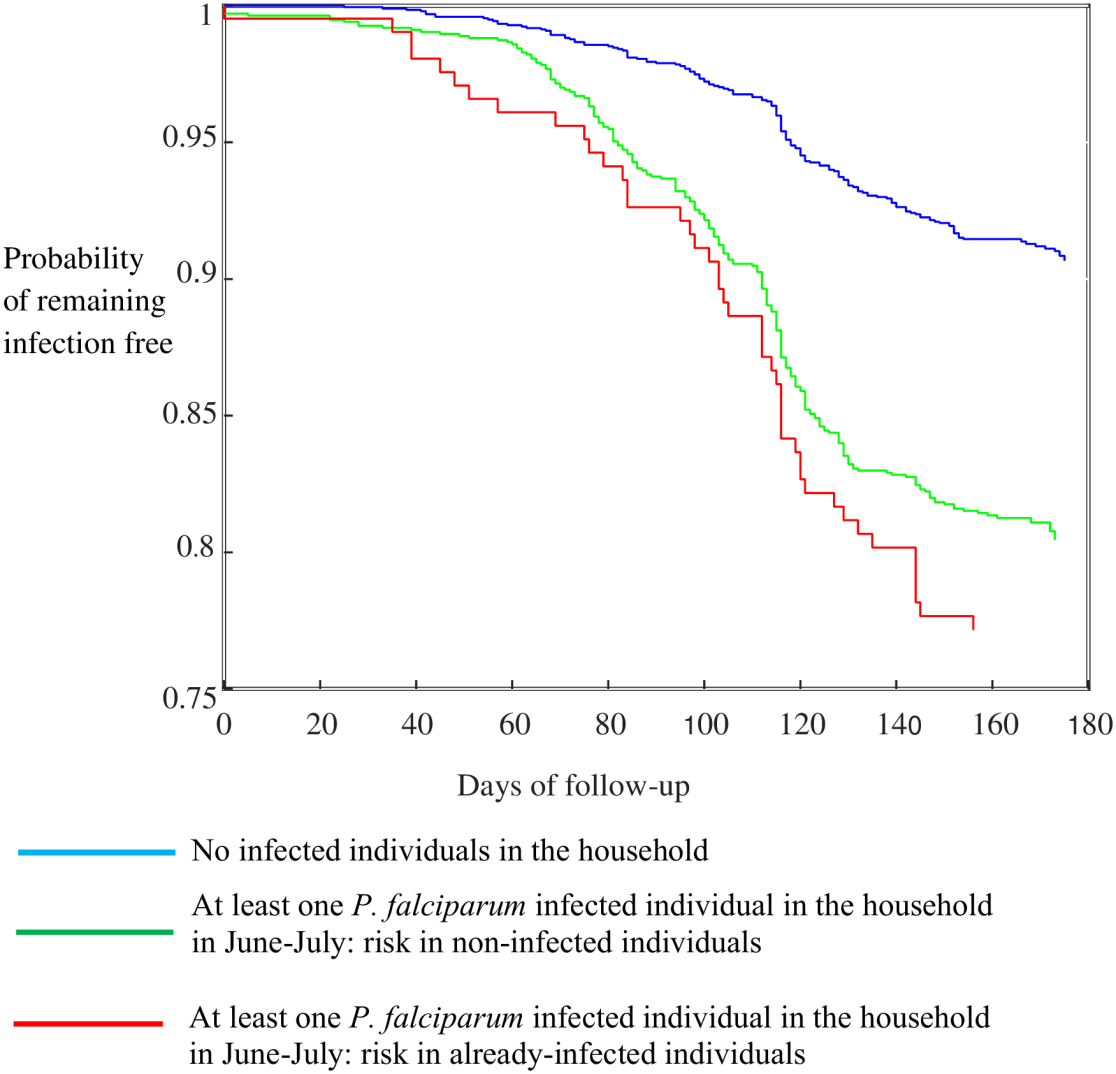
Kaplan Meier survival curves comparing the time to clinical malaria among household members with or without at least a malaria-infected individual at the start of the transmission season. After adjusting for age, gender, travel, LLIN use and sleeping outdoors, the risk remained significantly higher only for WCR (HR = 4.0, 95% CI: 2.1–7.5, p<0.01) and URR-N (HR 1.5, 95% CI: 1.1–2.1, p = 0.02).

In households with at least one malaria-infected individual at the beginning of the transmission season, the household risk of clinical malaria for other household members was significantly higher (HR = 1.76, p<0.01) than in the rest of the population, regardless of their infection status (uninfected: HR = 1.43, p<0.01; infected: HR = 1.76, p<0.01) (Fig 4)

A total of 10,184 anopheles mosquitoes were captured, almost half (45.24%, 4607/10184) by HLC. *An*. *gambiae s*.*l*. was the main vector in all regions, with the exception of CRR where *An*. *funestus* was the most predominant one (53.77%, 1865/3468). Indoor biting, after weighing for sleeping indoors or outdoors, was higher than outdoors biting, in all regions. Indoor biting was significantly higher in the WCR than in other regions (p<0.01) except in the CRR (p = 0.74) (Table 8).

**Table 8:** *An*. *gambiae s*.*l*. indoor and outdoor biting estimated by HLC, by region*.

Region	*An*. *gambiae s*.*l* indoor densities	*An*. *gambiae s*.*l* outdoor densities	Weighted proportion indoor biting	Weighted proportion outdoor biting	Indoor/outdoor biting	p value (compared to WCR)
WCR	532	817	87.72	12.28	7.14	
NBR	109	95	80.95	19.05	4.25	0.01
LRR	635	589	81.11	18.89	4.29	<0.01
CRR	327	329	88.20	11.80	7.48	0.74
URR-S	201	163	82.64	17.36	4.76	0.01
URR-N	214	202	76.61	23.39	3.28	<0.01

* Weighted proportions in indoor densities and outdoor densities adjusted for using the proportion of individuals sleeping indoors and outdoors

Indoor biting was significantly lower in July than in each of the following months, with more than 90% of indoor biting between August and November (Table 9).

**Table 9:** *An*. *gambiae s*.*l*. indoor and outdoor biting by month*.

Month	Indoor HLC densities	Outdoor HLC densities	Weighted proportion indoors	Weighted proportion outdoor	Indoor/outdoor ratio	p value (compared to July)
July	223	227	57.45	42.55	1.35	
August	226	322	94.20	5.80	16.25	<0.01
September	1230	1255	96.79	3.21	30.17	<0.01
October	271	334	90.85	9.15	9.93	<0.01
November	167	211	98.05	1.95	50.33	<0.01
December	81	59	82.85	17.15	4.83	0.02

* No vectors were captured by HLC in June

**Fig 5:**
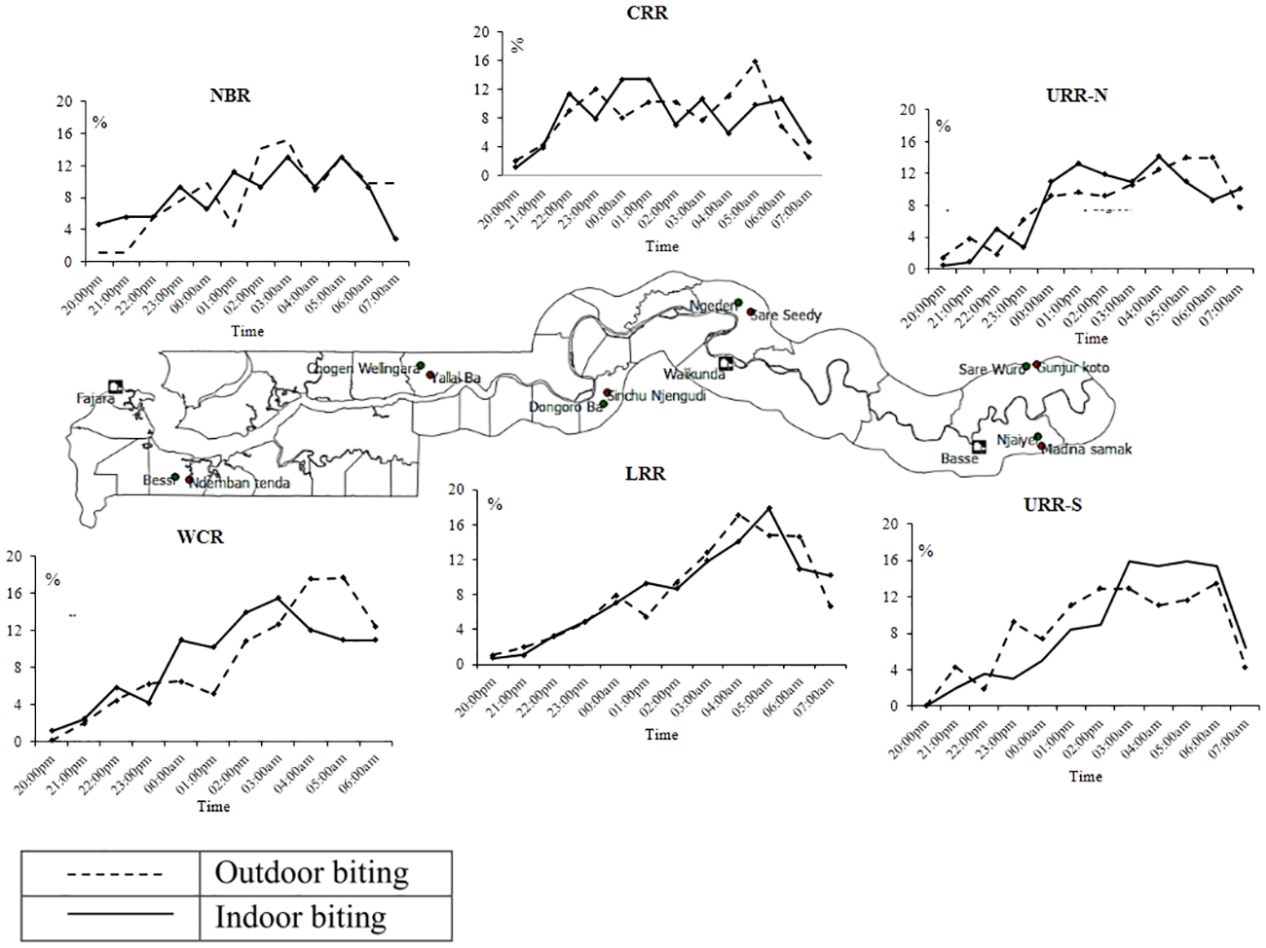
*An*. *gambiae s*.*l*. indoor and outdoor biting patterns across study sites.

A substantial proportion of biting occurred between 4-6am in all regions (Fig 5)

**Fig 6:**
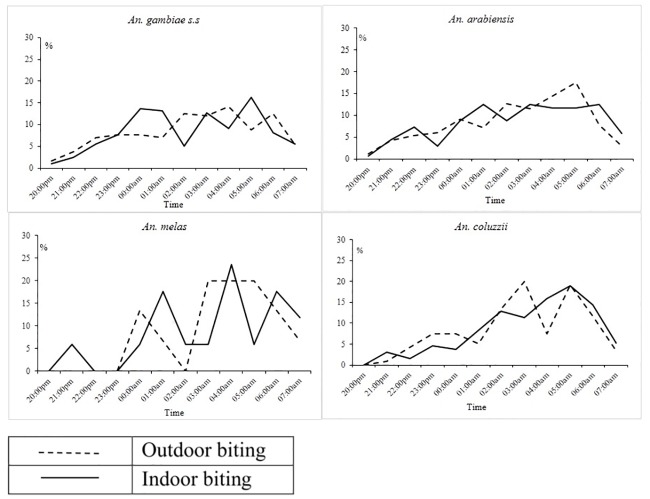
Indoor and outdoor biting patterns on *An*. *gambiae s*.*s*, *An*. *melas*, *An*. *coluzzii and An*.*arabiensis* during the 2013 transmission season.

Similarly for each vector species, substantial proportion of indoor and outdoor biting occurred between 4am-6am (Fig 6)

Speciation by PCR was successful for 65.43% (1963/3000) of the *An*. *gambiae s*.*l*. *An*. *coluzzii* was the predominant species in western (WCR and NBR) and eastern (URR-S and URR-N) Gambia while *An*. *gambiae s*.*s*. was most common in the other two regions, LRR and CRR, in the centre of The Gambia. *An arabiensis* was mainly found in the central and eastern regions (Table 10).

**Table 10:** Entomological inoculation rates (EIR) and species composition by region.

	EIR	Species composition (%)					
Region		*An*.*arabiensis*	*An*. *gambiae s*.*s*	M/S Hybrid	*An*. *melas*	*An*. *coluzzii*	*total*
WCR	0.14	90 (14.54)	89 (14.38)	98 (15.83)	39 (6.30)	303 (48.95)	619
NBR	0	26 (19.70)	18 (13.64)	10 (7.58)	8 (6.06)	70 (53.03)	132
LRR	4.73	206 (36.08)	272 (47.64)	10 (1.75)	63 (11.03)	20 (3.50)	571
CRR	2.33	132 (29.07)	313 (68.94)	6 (1.32)	1 (0.22)	2 (0.44)	454
URR-S	3.66	27 (28.13)	6 (6.25)	10 (10.42)	0	53 (55.21)	96
URR-N	3.26	35 (38.46)	10 (10.99)	9 (9.89)	0	37 (40.66)	91

**Fig 7:**
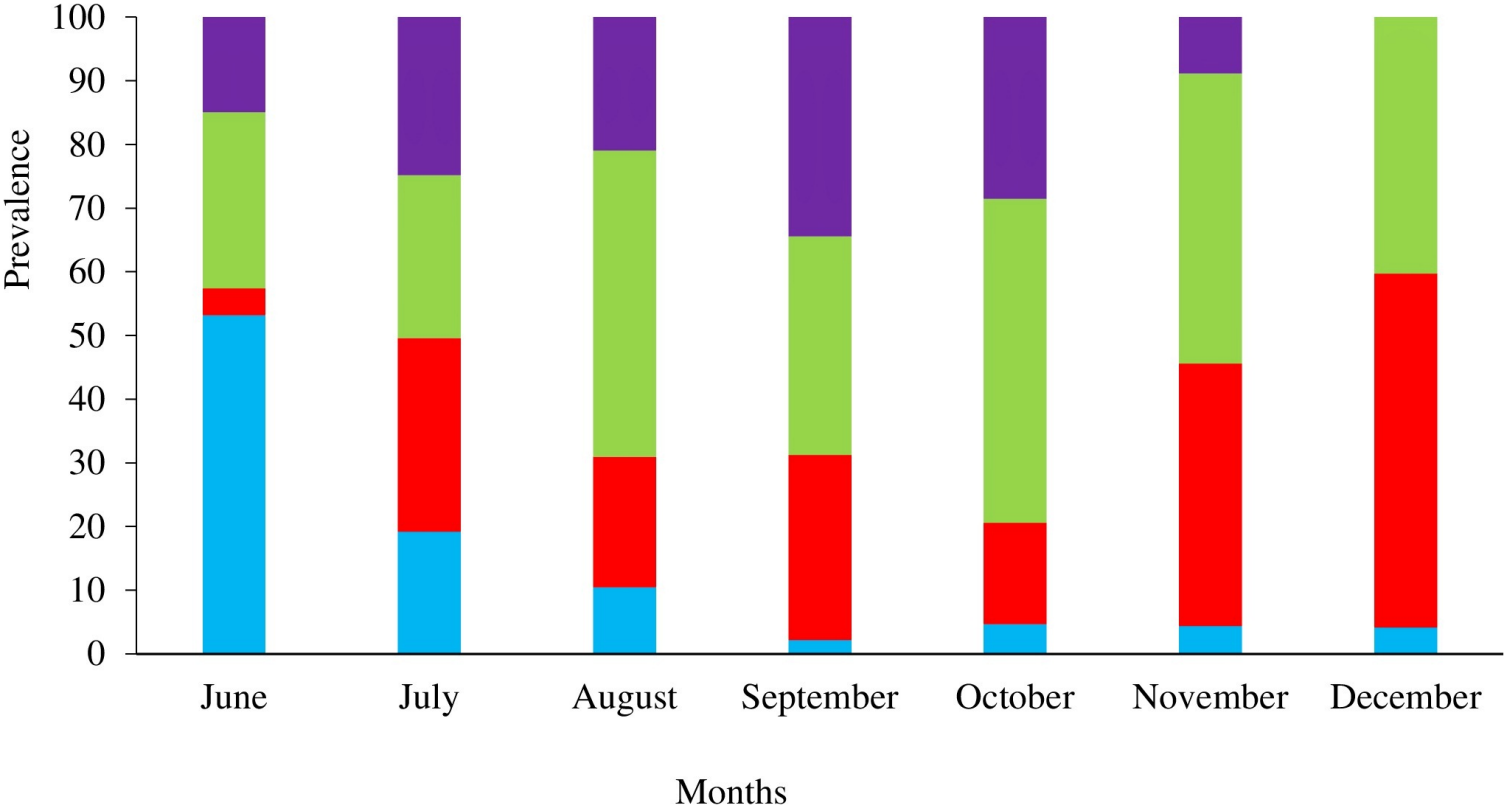
Variation *An*. *gambiae s*.*l* species composition across the transmission season. Vector parity was significantly higher in URR-S and URR-N than in the other 4 regions (Table 11).

The species composition varied substantially during the transmission season (Fig 7)

**Table 11:** Vector parity by region.

Region	Parous/Parous+ Nulliparous	Parity(95% CI)	p value
WCR	341/826	41.28 (37.93–44.64)	-
NBR	47/173	27.16 (20.54–33.79)	0.03
LRR	383/788	48.60 (45.11–52.09)	0.01
CRR	338/479	70.56 (66.48–74.65)	<0.01
URR-S	117/129	90.69 (85.68–95.71)	<0.01
URR-N	227/280	81.07 (76.48–85.65)	<0.01

*Comparison of parity between the WCR and other regions

**Fig 8:**
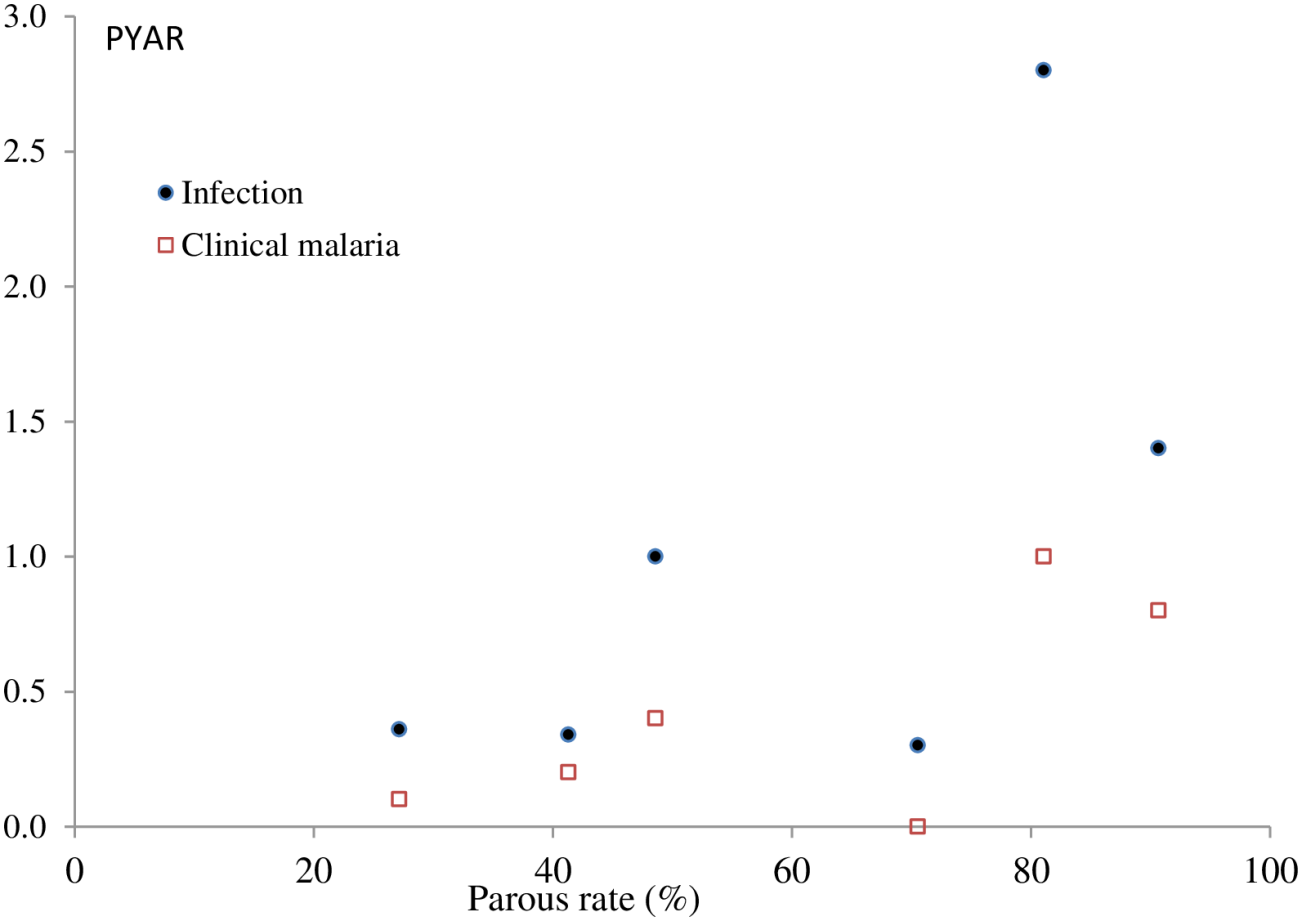
Relationship between parous rate (X axis) and incidence of infection and clinical malaria (Y axis). This was confirmed by a multivariate analysis (vector parity, EIR, and species variation) in which a percentage increase in vector parity was significantly associated with the incidence of infections (IRR: 7.11; 95%CI: 6.32–8.00) (p<0.01). Sporozoite prevalence was 0.14% (14/10184), with no positive samples in NBR. The EIR was similar in LRR (4.73/person/year), URR-S (3.66/person/year) and URR-N (3.26/person/year), the regions with the highest incidence of malaria infections though CRR, with the lowest incidence of infection, had an EIR of 2.33; EIR was extremely low in WCR (0.14/person/year) and NBR (0/person/year) (Table 10).

The unadjusted vector parity was correlated (Spearman) with the incidence of malaria infection (r^2^ 0.7, p<0.01) but not with that of clinical malaria (r^2^ 0.4, p = 0.3) (Fig 8)

## Discussion

The prevalence and incidence of malaria infection and clinical disease during the 2013 transmission season were significantly different across the six study sites, despite similar high coverage of control interventions. Mass distribution of LLINs was carried out in 2009, 2011 and 2014, and annual indoor residual spraying (IRS) with DDT since 2009; the last cycle was implemented in August 2013 in all rural regions [36]. The most striking difference between regions, possibly explaining the higher endemicity in the eastern part of the country, was the high vector parous rate in URR, indicating high survival rate which would increase the vector’s probability of becoming infectious. Such high vector survival is surprising when considering the high coverage of LLINs and IRS. In 2008, DDT and permethrin resistance was reported in western but not eastern Gambia [37]. However, a few years later, high resistance to pyrethroids and DDT was observed in one village in URR-S.[38] In 2013, it was confirmed that phenotypic resistance to DDT (mortality-*An*. *gambiae s*.*s*. 6%; An. arabiensis 9%; *An*. *coluzzii* 67%) and deltametrin (mortality-*An*. *gambiae s*.*s*. 41%; *An*. *arabiensis*: 97%; *An*. *coluzzii*: 89%) was more common in eastern Gambia [39]. However, a recent multi-country study did not find any association between malaria incidence and insecticide resistance [40].

Differences in species composition between and within the different regions may further explain the observed residual transmission and high endemicity in the eastern region. *An*. *arabiensis* and *An*. *coluzzii* represented together about 80% of the vector population in URR, where the incidence of infection and clinical malaria was the highest. These two species are well adapted to the semi-arid conditions found in the eastern region and are highly efficient at transmitting infection during the short transmission season. *An*. *coluzzii* is anthropophilic, endophilic, endophagic, and late-night biting [18] while *An*. *arabiensis* is more zoophilic, exophily, exophaghic, and early-night biting [12], though in our study these two species had similar biting patterns. The low presence of *An*. *gambiae s*.*s* in the URR may be the result of out-competition from other species. Entomological surveys in Gambia have shown heterogeneity in *An*. *gambiae s*.*l* species composition [41, 42] with the more recent survey showing heterogeneity between and within the village pairs [43]. Therefore, the presence of outdoor biting species or indoor feeding species with exophilic behavior suggests that, despite high coverage of vector control interventions, they can still escape the insecticides’ killing or excito-repellent action [12]. The higher risk for infection associated with sleeping outdoors and with traveling outside the village, when people are less likely to use protective measures, suggests outdoor biting probably contributes to the on-going transmission. Besides physiological insecticide resistance, behavioural resistance should also be considered. In LRR, URR north and south there is still substantial biting activity early morning, between 4am and 6am. As substantial early morning biting, both indoor and outdoor, had already been described in 1992 in central Gambia,[44] a behaviour change in response to the high LLIN coverage is unlikely. The higher malaria burden in eastern Gambia compared to the rest of the country had previously been observed in the early 1990s. This region also had also the lowest vector densities and it was hypothesized that the higher malaria prevalence was due to the low mosquito nuisance which in turn resulted in low LLIN use [10, 45]. Such hypothesis does not seem plausible given the current high LLIN coverage. Other unidentified factors may be responsible for the observed heterogeneity of transmission and the decreasing gradient of malaria transmission, from east to west, such as differences in human behaviour leading to higher exposure; for example, in URR the large majority of people who slept outdoors did not use LLINs while in the other regions LLINs were used also outdoors.

Malaria prevalence in the dry season was similar to that observed at the beginning of the 2013 transmission season. However, considering that most individuals infected in April 2014 were malaria negative in December 2013, their infection was probably acquired at the very end of the transmission season, when incidence of infection was the highest; thus most infections identified in December were either cleared over the next 2–3 months or under the detection threshold in April [46]. Individuals infected at the very end of the transmission season carry their infections for six months during the dry season [22, 24, 47], and re-start transmission by infecting the vector after the onset of rains.

Though malaria prevalence was strikingly similar in June 2013 (5.0%) and April 2014 (6.7%), gametocyte carriage at these two time points was extremely different, 0% and 14%, respectively. It is unclear whether individuals infected in April 2014, who possibly restart malaria transmission at the onset of rains, would gradually clear gametocytes. In the 2003 dry season, in Farafenni, about half of the 37 infections detected carried gametocytes; interestingly the proportion of infections with gametocytes did not vary by season though infectivity to mosquitoes varied [48]. Seasonal variation of infectiousness has been recently confirmed; in Burkina Faso, infectiousness was the highest (48.1%) at the onset of the transmissions season and declined steadily to 15.5%, just at the beginning of the dry season. This may indicate some stimulation for the production of gametocytes in asymptomatic individuals or an immunity-related phenomenon favouring transmission to mosquitoes [49]. Higher infectiousness at the beginning of the transmission season fits with the trends of incidence and prevalence observed in most of our villages, particularly in eastern Gambia.

The risk of malaria infection at the start of the transmission season was higher among individuals who had travelled outside the study villages. This highlights the importance of human mobility in maintaining transmission, especially in settings aiming at achieving pre-elimination [50]. Women had a lower risk of infection than men, an observation that may be due to the use of control interventions as females are more likely to use LLINs than males as previously reported in Uganda, Tanzania and Angola [51].

The risk of clinical malaria was higher in households with at least one malaria-infected individual at the beginning of the transmission season. Though this association, after adjusting for several confounding factors, remained significant only in two regions, it probably indicates the higher risk of exposure to malaria in these households, possibly because of their location in relation to breeding sites or their structure. Such clustering of malaria cases has been previously reported in a pooled analysis, showing that household members of index malaria cases were overall five-times more likely to have malaria than members of other households [52]. In Kenya, there was clustering of febrile malaria cases at individual household level [50]. Identifying such infected individuals and treating the whole household before the transmission season could be an alternative approach to mass drug administration. Pending the availability of diagnostic tests able to detect low density infections, this could delay the spread of infection across the communities during the transmissions season and thus reduce the number of infected individuals at the end of the season.

Older children were at higher risk of multiple episodes of malaria infections, an observation already made in settings with low malaria transmission. [53, 54] Studies in southern Africa also report higher odds of asymptomatic infections in school-age children compared to younger children [53]. As transmission decreases, exposure to infection in the early years of life also decreases, delaying the acquisition of antimalarial immunity [55, 56]. In addition, older children have a larger body surface area and tend to stay out at night longer than younger children and are thus more likely to be exposed to infection [12]. The short and long term consequences of multiple infections in older children cannot be understated. Indeed chronic asymptomatic malaria infections are associated with recurrent episodes of mild to moderate anaemia, co-infection with invasive bacterial disease especially non-typhoid salmonellae, [57] and on-going malaria transmission [54]. In such settings, targeting school-age children with malaria control interventions may reduce substantially the human reservoir of infection.

The EIR is considered a standard metric of malaria transmission. [58] However, its precision and accuracy are usually low because of the intrinsic difficulties of obtaining robust measures of human biting rate and particularly sporozoite rate, due largely to the highly variable nature of the mosquito population, particularly where vector density is low [58] as in eastern Gambia. Indeed, EIR was particularly low in NBR and WCR, but varied only between 3 to 4/person/year in all other regions despite marked differences in the force of infection. This was expected as EIR estimates the rate of human exposure to infectious bites and does not directly translate into population measures of incidence of infection or clinical disease. [58] Though HLC can be used to determine human biting frequency and biting time by vector species [59], the method has important limitations because it can be performed in a limited number of houses, in our study two houses per village were selected, which may not be representative of the whole village, and collections are carried out by unprotected volunteers, which provide figures of vector biting that do not reflect the level of exposure of individuals protected by LLIN. Comparisons of the indoors and outdoors biting rates is limited as the study did not collect information on the actual time study subjects went to bed or woke up. Previous studies have adjusted the hourly vector densities by the hourly proportions of individuals who slept indoors.[60, 61] However, in our study, more than 65% of individuals who reported sleeping outdoors used a LLIN while almost 20% of those who reported sleeping indoors did not use a LLIN. Therefore, exposure to infective bites in the early morning and late evening should be interpreted with caution. Indoor and outdoor biting rates were adjusted on the basis of sleeping habits (indoors-outdoors) and results should be considered as grossly indicative of the risk of infection.

The follow up of the study population was intense (monthly bleeding of all inhabitants in the study villages for seven consecutive months) and challenging. Some groups were more difficult to follow up than others, for example adult males were more often traveling than females and children, and this could have affected some risk estimates. However, the overall loss to follow up was relatively low (11.0%) given the study design and unlikely to significantly affect the study outcomes.

In conclusion, there is still significant residual malaria transmission across The Gambia, particularly in the eastern region. This is maintained by a low-density vector population with high survival, despite the high coverage of conventional vector control interventions. Even in sites where transmission is significantly lower than in eastern Gambia, a non-negligible proportion of individuals maintain malaria infections, from the previous transmission season to the next one. Such individuals should be identified and targeted with additional interventions aiming at reducing the human reservoir of infection. Additionally, new interventions able to target vectors escaping LLIN and IRS are needed.

## Supporting information

S1 Table Proportion of infections with gametocyte by month. (DOCX)

S1 File Case report form. (PDF)
