# Supplementary material for: Residual malaria transmission dynamics varies across The Gambia despite high coverage of control interventions
Source: PLoS One. 2017 Nov 2;12(11):e0187059. doi: 10.1371/journal.pone.0187059 (PMC5667860; doi:10.1371/journal.pone.0187059)
Supplement: S1 Table — (DOCX) [file pone.0187059.s001.docx]

| **Month** | **Proportion of infections with**  **gametocytes (n/N)** | **95% CI** | **p-value** |
| --- | --- | --- | --- |
| June | 0.0 (0/70) | 0 |  |
| July* | 9.39 (17/181) | 5.14-13.64 |  |
| August | 7.11 (14/197) | 3.52-10.69 | 0.37 |
| September | 12.98 (20/154) | 7.68-18.29 | 0.25 |
| October | 18.31 (65/355) | 14.29-22.33 | 0.007 |
| November | 21.69 (113/521) | 18.15-25.23 | <0.01 |
| December | 13.44 (48/357) | 9.91-16.98 | 0.14 |
| April-14 | 14.09 (31/220) | 9.49-18.68 | 0.13 |

*Comparisons of proportions made to July

#### Supplementary Table 1. Proportion of infections with gametocyte by month
