# Supplementary material for: Residual malaria transmission dynamics varies across The Gambia despite high coverage of control interventions
Source: PLoS One. 2017 Nov 2;12(11):e0187059. doi: 10.1371/journal.pone.0187059 (PMC5667860; doi:10.1371/journal.pone.0187059)
Supplement: S1 File — (PDF) [file pone.0187059.s002.pdf]

SUPPORTING INFORMATION

SCC 1318 MALARIA TRANSMISION DYNAMICS IN GAMBIA

MRC The Gambia Unit

SERIAL NUMBER|_|_|_|_|_|_

PARTICIPANT MONTHLY CASE REPORT FORM (2013)

**GENERAL INFORMATION**	
1	Date of visit: |___|___|/|___|___|/|___|___|___|___| (DD/MM/YYYY)
2	Study ID: |___|___|___|___|___|___|___|___| (e.g. A00200101)
**MALARIA**	
3	Have you had a fever in the last month? 1- Yes 2- No 3- Don' t know **(If No skip to Q 8)**
4	Did you do a RDT? 1- Yes 0- No Don't know (if No go to Q6)
5	What was the RDT result 1- Positive 2- Negative 3- Don't know
6a	Where did you receive treatment for your fever?<br>1- Government hospital 2- Private clinic/hospital 3- Pharmacy 4- VHW 5 - Mobile clinic<br>6- Shop 7- Traditional healer 8- Marabou 9- Friends/family<br>10- No treatment 11- Other specify ____________________________
6b	Did you consult anyone else for your illness (such as another health worker, or marabout or someone else for your health? 1- Yes 2-No
7	What antimalarial medication did you receive? **(Tick all that apply)**<br>1- Coartem 2- Fansidar 3- Chloroquine 4- Other specify__________________<br>5 - Don't know
8	Have you had a fever in the last 24 hours? 1- Yes 0- No **(If No skip to Q 10)**
**If history of fever in the last 24 hours perform an RDT**	
9	RDT result 1- Positive 2- Negative 3- Don't know
**BEDNET USE**	
10	Did you sleep under your bed net
A	Last night 1- Yes 0- No
B	The night before last 1- Yes 0- No
11	In the past 1 month have you sometimes slept outside the house?<br>1 - Yes 0 - No
12	If yes, did you use a bed net when you slept outside?<br>1 - Yes 0 - No
13	In the last 1 month have you travelled out of the village? e.g. cultivating the fields, herding cattle, or during other work activities? **(If No skip to Q 16)**<br>1- Yes 0- No
14	If Yes how many days did you stay outside the village? |____|____| (days)

15	Did you sleep under a bed net when you stayed outside the village?<br>1- Yes 0- No		
**PHYSICAL EXAMINATION**			
16	Axillary Temperature |____|____|.|____|° C<br>(***If axillary temperature 37.5˚C or more, perform a RDT)***		
17	RDT result? 1- Positive 2-Negative		
**SAMPLE COLLECTION,**<br>***(always take a blood slide, collect 3 blood spots samples on filter paper and check haemoglobin*****)**			
		**Sample label Unique ID**<br>(Village letter| Compound No.| HH No.| Collection Time point (A-G)| Year (e.g. C0010101A1)	
18	Thick blood smear collected on slide	1- Yes 0- No	Record Sample Label ID<br>|__|__|__|__|__|__|__|__|__|__|
19	Three (3) dried blood spots collected?	1- Yes 0- No	|__|__|__|__|__|__|__|__|__|__|
20	Hemoglobin result	|____|____|.|____| g/dl	

Nurse Field Workers Name:___________________________________ Initials: |___|___|

Date: |___|___|/|___|___|/|___|___|___|___|
